# Adjuvant Interstitial Yttrium-90 Hydrogel (IsoPet^®^) Following Limb-Sparing Marginal Excision of Multifocal Distal Pelvic-Limb Neoplasia in a Dog

**DOI:** 10.3390/ani16213452

**Published:** 2026-11-01

**Authors:** Goutham Kumar Isai, Kevin A. Drygas, Brad Weeks

**Affiliations:** 1 Capital Veterinary Specialists LLC, 1615 Mahan Center Boulevard, Tallahassee, FL 32308, USA; kevind33@aol.com; 2 Vivos Inc., Suite N288, 1030 N. Center Parkway, Kennewick, WA 99336, USA; brad.weeks@vivosinc.com

**Keywords:** canine, neoplasia, soft tissue tumor, incomplete histologic margins, yttrium-90, IsoPet^®^, precision radionuclide therapy, brachytherapy, veterinary oncology

## Abstract

Background: Achieving complete surgical margins can be challenging for tumors involving the distal extremities due to limited surrounding soft tissue and the proximity of functionally important structures. Incompletely excised tumors may require adjuvant radiation therapy to treat suspected residual microscopic disease. Yttrium-90 phosphate hydrogel (IsoPet^®^) is an emerging injectable radiation modality that delivers localized beta radiation to a tumor bed or surgical margin. Case Description: An eight-year-old neutered male Bullmastiff was evaluated by the referring veterinarian for firm subcutaneous masses developed along the right pelvic limb. Fine-needle aspiration was initially suggestive of an inflammatory process; however, subsequent punch biopsies identified Grade II soft tissue sarcoma. The patient was referred for surgical oncology evaluation. Four right pelvic limb masses and the right popliteal lymph node were surgically excised. Histopathological examination of the excisional specimens identified poorly differentiated carcinoma involving the excised masses, with metastatic involvement of the right popliteal lymph node and incomplete surgical margins. Adjuvant Y-90 hydrogel was elected following complete surgical wound healing. On 20 November 2025, 8.46 mCi (313 MBq) of Y-90 phosphate hydrogel in a total volume of 4.7 mL was administered through 66 peri-incisional and incisional injections distributed across the previous right pelvic limb surgical beds using a grid-based technique. The procedure was completed without complications. Post-treatment follow-up demonstrated satisfactory wound healing, with only mild transient appetite reduction and minimal localized tissue irritation. After surgery plus adjuvant Y-90, no clinically detectable local recurrence was identified at the treatment sites during the 120-day documented follow-up period. Seventy-four days following Y-90 hydrogel treatment, computed tomography performed for persistent otic disease identified an aggressive osteolytic lesion involving the right C5 vertebra with extradural spinal cord compression. The relationship between this lesion and the previously treated pelvic limb neoplasia could not be definitively established. Despite the subsequent development of this cervical lesion, no clinically detectable recurrence was documented at the original treatment limb sites. This finding is an observation after combined surgery and Y-90, not evidence of Y-90-specific efficacy. The patient was subsequently managed supportively and was euthanized by the referring veterinarian approximately four months after identification of the cervical lesion. Conclusion: This single case demonstrates that peri-incisional Y-90 phosphate hydrogel could be administered to multiple distal pelvic-limb surgical beds after limb-sparing excision. The procedure was well tolerated. No clinically detectable local recurrence was identified at the treated sites during the 120-day documented follow-up period. Because gross disease had already been removed and there was no comparator, these observations cannot establish the independent efficacy of IsoPet^®^. Prospective studies are needed.

## Simple Summary

Achieving complete surgical margins can be challenging for tumors involving the distal extremities because limited surrounding soft tissue and the proximity of functionally important structures may restrict wide surgical excision. This case describes an eight-year-old neutered male Bullmastiff with multifocal neoplasia involving the right pelvic limb. Four right pelvic limb masses and the right popliteal lymph node were surgically excised using a limb-sparing approach. Histopathological examination identified poorly differentiated carcinoma involving all four excised masses, metastatic involvement of the right popliteal lymph node, and incomplete surgical margins. Following postoperative wound healing, yttrium-90 phosphate hydrogel was administered through multiple peri-incisional injections distributed across the previous surgical beds. There were no complications during the treatment. After combined limb-sparing surgery and adjuvant Y-90, follow-up examinations demonstrated satisfactory wound healing. No clinically detectable local recurrence was observed at the treatment sites during the 120-day documented follow-up period. These observations cannot establish the independent efficacy of IsoPet^®^.

## 1. Introduction

Soft tissue neoplasms are commonly encountered cutaneous and subcutaneous tumors in dogs and comprise a heterogeneous group of tumors with variable biological behavior. Surgical excision remains the principal treatment for localized disease, with complete histologic margins being an important determinant of local tumor control. In a systematic review and meta-analysis of canine cutaneous and subcutaneous soft tissue sarcomas, local recurrence was reported more frequently following incomplete excision than complete excision, emphasizing the importance of achieving adequate surgical margins whenever anatomically possible [1].

Tumors involving the distal extremities present a particular challenge because limited surrounding soft tissue and the proximity of joints, tendons, and neurovascular structures may restrict the ability to obtain wide surgical margins without compromising limb function. Consequently, limb-sparing surgery combined with adjuvant treatment may provide an alternative to amputation in selected patients. Postoperative radiation therapy is an established adjunctive treatment for incompletely excised canine soft tissue sarcomas and has been associated with prolonged local control [2,3]. In a retrospective study of 35 dogs with incompletely excised soft tissue sarcomas, postoperative radiation therapy was associated with a median time to local recurrence exceeding 798 days [2].

Although conventional external beam radiation therapy can provide effective local treatment, it generally requires multiple treatment fractions and therefore repeated visits and anesthetic episodes. These practical considerations have encouraged investigation of alternative radiation strategies, including hypofractionated and single-fraction protocols, particularly for patients in which minimizing treatment burden is important [4,5].

IsoPet^®^ Precision Radionuclide Therapy (PRnT™) is a single localized option that may be considered when multiple distal surgical beds make fractional external beam radiation impractical. The therapy utilizes yttrium-90 (^90^Y) phosphate particles incorporated within an injectable hydrogel, which can be administered directly into a tumor or surgical bed. Y-90 is a high-energy beta-emitting radionuclide, and its relatively short tissue penetration allows radiation to be concentrated within and around the treatment site. The Y-90-polymer system was developed to provide localized, high-dose beta radiation while limiting radiation exposure to surrounding tissues [6,7].

The localization of the radioactive material is an important feature of this treatment approach. Experimental and clinical investigations of Y-90–polymer composites have evaluated radiation safety, biodistribution, and retention of the radioactive material following direct intratumoral administration. These studies support the ability of the injectable Y-90–polymer system to deliver radiation locally while minimizing redistribution to distant tissues [6,7].

More specifically, ^90^Y-IsoPet^®^ has been evaluated in naturally occurring canine soft tissue sarcomas. Ref. [8] retrospectively evaluated six canine cases treated with intratumoral ^90^Y-IsoPet^®^ and investigated the spatial distribution of the injected hydrogel and absorbed radiation dose using imaging-based and Monte Carlo dosimetric methods. A grid-based injection pattern was used to achieve relatively uniform dose distribution within the clinical target volume. Their findings provide important dosimetric information for treatment planning and demonstrate the feasibility of characterizing radiation distribution following IsoPet^®^ administration in canine soft tissue sarcomas [8]. Those dosimetric methods informed the grid-based peri-incisional technique used here; they are cited as technical guidance rather than as evidence that poorly differentiated carcinoma will respond in the same way as sarcoma.

The potential clinical advantage of IsoPet^®^ is particularly relevant for tumors in anatomically challenging locations. Conventional postoperative radiation requires repeated treatment sessions, whereas localized Y-90 radionuclide therapy can potentially deliver a high radiation dose directly to a defined tumor or surgical bed during a single treatment procedure. This approach may therefore be useful when preservation of limb function is prioritized and conventional wide surgical excision is difficult. However, the clinical evidence for IsoPet^®^ remains limited compared with the substantially larger evidence base supporting conventional radiation therapy. The current literature includes radiation-safety studies and emerging canine dosimetric and clinical investigations, emphasizing the need for further clinical evaluation of this modality [6,7,8].

The definitive diagnosis in this case was poorly differentiated carcinoma rather than soft tissue sarcoma. Canine apocrine sweat gland adenocarcinomas are uncommon cutaneous neoplasms that may exhibit local invasion and metastatic potential [9,10]. Clinical reports have also documented local recurrence and subsequent distant metastatic disease in canine apocrine sweat gland adenocarcinoma, highlighting the malignant and potentially metastatic behavior of these tumors [11]. The management of incompletely excised soft tissue sarcomas provides relevant clinical context for postoperative local treatment when complete margins cannot be achieved [12,13,14,15], although the biological behavior and treatment response of poorly differentiated carcinoma may differ from those of soft tissue sarcoma. Thus, the soft tissue sarcoma literature provides a rationale for considering postoperative local treatment in the setting of incomplete margins but does not establish the efficacy of Y-90 hydrogel for carcinoma.

The present report describes the management of an eight-year-old neutered male Bullmastiff with poorly differentiated carcinoma involving the right pelvic limb. Following referral for specialty care, four right pelvic limb masses and the regional popliteal lymph node were surgically excised using a limb-sparing approach. Histopathological examination of the excisional specimens identified poorly differentiated carcinoma with regional lymph node metastasis and incomplete surgical margins. Because residual microscopic disease was suspected, adjuvant Y-90 hydrogel was administered following postoperative wound healing. Treatment consisted of 8.46 mCi (313 MBq) of Y-90 phosphate hydrogel delivered through 66 peri-incisional injections. This report describes the clinical decision-making, limb-sparing surgical management, IsoPet^®^ treatment, postoperative tolerance, whether clinically detectable local recurrence was observed at the treated sites, and subsequent clinical events.

## 2. Case Presentation

### 2.1. Signalment and Initial Presentation

An 8-year-old neutered male Bullmastiff weighing 67.1 kg (148 lb) was referred to the Surgical Oncology Service at Capital Veterinary Specialists, Tallahassee, Florida, for evaluation and management of multiple soft tissue masses involving the right pelvic limb.

To further investigate the orthopedic abnormalities, pelvic and stifle radiographs were obtained while the patient was sedated. Mild degenerative changes in the right hip were identified, whereas the right stifle was radiographically unremarkable. No aggressive osseous lesion or soft tissue abnormality was recognized at that time. Based on the clinical and radiographic findings, the patient was diagnosed with degenerative joint disease, and conservative management consisting of anti-inflammatory medication and monthly bedinvetmab (Librela^®^) injections were initiated.

On 15 August 2025, the pet presented for a new swelling near the right hock. Physical examination revealed multiple firm, freely movable subcutaneous nodules extending from the medial aspect of the right tarsus proximally toward the medial thigh. Fine-needle aspiration of the masses was performed. Cytologic evaluation demonstrated predominantly macrophages and neutrophils, findings considered most consistent with an inflammatory process; however, the possibility of neoplasia could not be excluded because of the low cellularity of the samples. The patient was treated empirically with antibiotics and prednisolone, with recommendation for biopsy if the lesions failed to regress.

Despite medical therapy, the masses continued to enlarge over the following several weeks. Progressive enlargement of previously identified nodules was noted with additional masses noted along the medial aspect of the right pelvic limb several weeks later. Because of continued progression despite appropriate medical management, inflammatory disease became less likely, and tissue biopsy was recommended.

On 29 September 2025, three representative punch biopsy samples were obtained from the following areas:

Caudal lateral aspect inside right rear leg, 8 mm.

Medial lump inside right rear leg, marked with suture, 6 mm.

Proximal to the right hock, 8 mm.

Histopathological examination demonstrated Grade II soft tissue sarcoma in two of the submitted specimens, while another lesion was diagnosed as a hybrid follicular cyst. Given the multifocal nature of the disease and the anticipated complexity of surgical management, the patient was referred to Capital Veterinary Specialists for definitive oncologic evaluation and treatment.

### 2.2. Referral Evaluation, Surgical Planning, and Limb-Sparing Surgery

Following histopathological confirmation of Grade II soft tissue sarcoma by the referring veterinarian, the pet was referred to the Surgical Oncology Service at Capital Veterinary Specialists (CVS), Tallahassee, Florida, for definitive treatment. At the time of referral, the patient had a several-month history of progressive enlargement of multiple masses affecting the right pelvic limb despite conservative medical management. The owners reported that the lesions had continued to increase in size, although the patient remained ambulatory with an acceptable quality of life.

The referral consultation involved a comprehensive review of the patient’s medical history, prior diagnostic workup, cytology results, and punch biopsy histopathology. Particular consideration was given to the multifocal nature of the lesions and their anatomical location in the distal pelvic limb, as these characteristics were anticipated to affect surgical planning and postoperative management.

Physical examination confirmed the presence of multiple discrete subcutaneous masses involving the right pelvic limb. The largest lesion was located over the caudal aspect of the right hock and measured approximately 3 cm × 3 cm × 3 cm, with superficial ulceration of the overlying skin. Additional firm, freely movable masses were identified over the cranial aspect of the tibia, the medial distal thigh (approximately 0.4 cm × 3 cm), and the medial proximal thigh (1.5 cm × 1 cm × 0.5 cm). No significant abnormalities were detected on general physical examination, and no additional cutaneous masses were identified elsewhere on the body.

The multifocal distribution of the lesions presented a considerable surgical challenge. Wide surgical excision with conventional oncologic margins was considered unlikely to be achievable because of the limited amount of surrounding soft tissue, the proximity of tendons and joints, and the need to preserve limb function. Consequently, several treatment options were discussed with the owners, including limb amputation, limb-sparing excision, and multimodal treatment incorporating postoperative radiation therapy.

Although limb amputation was expected to provide the greatest likelihood of complete local disease control, the owners expressed a strong preference for preserving limb function. After discussion of the anticipated benefits and limitations of each treatment option, a limb-sparing approach was elected. Because complete histological margins were considered unlikely to be achieved, consultation with the Radiation Oncology Service was obtained before surgery to develop an integrated treatment plan.

The Radiation Oncology team concurred that postoperative adjuvant radiation therapy would likely be indicated if microscopic residual disease remained following surgical excision. Rather than planning conventional external beam radiation therapy, the patient was considered a suitable candidate for IsoPet^®^ Precision Radionuclide Therapy (PRnT™) following complete wound healing, should histopathology confirm incomplete margins. This approach was chosen for the localized treatment of residual microscopic disease without the need for repeated anesthetic episodes that are associated with fractionated external beam radiation therapy.

## 3. Surgical Management and Histopathological Findings

The decision was made to proceed with limb-sparing surgical excision of all grossly visible tumors with histopathological assessment to determine the need for adjuvant treatment.

On 30 October 2025, the patient was anesthetized for definitive surgical management. Four separate masses involving the right pelvic limb were excised, including lesions from the right lateral cranial tibial region, right caudal hock, right medial distal thigh, and right medial proximal thigh. Because of concern for possible regional metastatic disease, the right popliteal lymph node was also excised and submitted for histopathological examination. The surgical procedure was completed without intraoperative complications, and the patient recovered uneventfully from anesthesia. Three-view orthogonal thoracic radiographs were obtained as part of the oncologic staging evaluation. No evidence of metastatic disease was identified, and the extra thoracic structures were unremarkable. Dedicated abdominal imaging, evaluation or sampling of additional regional lymph nodes, and whole-body CT staging were not performed before surgery or Y-90 treatment. No additional cutaneous masses were identified on physical examination, and no other clinically evident primary tumor was identified. Given the multifocal disease, incomplete surgical margins, and confirmed regional lymph-node metastasis, the treatment objective was local disease management and limb preservation following excision of the grossly visible right pelvic-limb masses. Systemic treatment was considered but was not elected. The right popliteal lymph node was completely excised, and histopathological examination confirmed metastatic poorly differentiated carcinoma. The surgical bed of the excised lymph node was not included in the subsequent Y-90 hydrogel treatment field.

Postoperative analgesia and antimicrobial therapy consisted of carprofen (150 mg orally every 12 h for 10 days), gabapentin (600 mg orally every 12 h as needed for pain), and cefpodoxime (600 mg orally every 24 h for 7 days), together with exercise restriction and routine wound care. Histopathological examination of the excised tissues identified poorly differentiated carcinoma involving all four excised masses, while the right popliteal lymph node contained metastatic poorly differentiated carcinoma. The reporting pathologist considered poorly differentiated apocrine gland carcinoma to be the most likely diagnosis; however, the exact tissue of origin could not be definitively determined on routine histopathology. The initial punch biopsies were obtained from three sites within the clinically affected pelvic limb; however, because the site descriptions in the referring records were limited and four separate masses were subsequently excised, precise one-to-one correspondence between each initial biopsy specimen and each excised lesion could not be established. This included the specimen initially diagnosed as a hybrid follicular cyst, for which definitive correspondence with a specific subsequently excised mass could not be determined. The initial punch-biopsy specimens and the final excisional specimens were evaluated by different diagnostic laboratory/pathologists and were not reviewed concurrently by the same pathologist. Histopathological examination of the excisional specimens demonstrated densely cellular, moderately demarcated, unencapsulated and infiltrative neoplasms composed of polygonal cells with distinct cell borders arranged in nests and islands on fine fibrovascular stroma. The cells had moderate eosinophilic cytoplasm, round centrally located nuclei with finely stippled chromatin, prominent nucleoli, moderate anisocytosis and anisokaryosis, and occasional multinucleation, karyomegaly and bizarre mitotic figures. Scattered necrosis and hemorrhage were also present, with variable mitotic activity. Comparable morphology was identified in the metastatic right popliteal lymph node. Immunohistochemical characterization was not performed. Although archived tissue was available, immunohistochemistry was not pursued because of cost considerations and because additional characterization was not expected to alter the treatment plan. Given the larger tissue volume and more extensive lesion architecture available in the excisional specimens, these findings were used for subsequent treatment planning. Marginal assessment showed complete excision of the right lateral cranial tibial mass (narrowest circumferential margin, 0.13 mm) and right medial proximal thigh mass (narrowest lateral margin, 0.19 mm; narrowest deep margin, 0.85 mm) but incomplete margins in the right medial distal thigh mass, where neoplastic cells extended through the inked circumferential surgical margin, and the right caudal hock lesion, where neoplastic cells extended through the inked and tagged distal surgical margin, indicating residual microscopic disease could not be ruled out. The initial punch biopsy diagnosis of Grade II soft tissue sarcoma differed from the definitive diagnosis of poorly differentiated carcinoma in the excisional specimens. Although the right cranial tibial and right medial proximal thigh masses were classified as completely excised histologically, their very narrow surgical margins raised concern about potential residual microscopic disease. Given the multifocal nature of the neoplasia, these sites were also included in the adjuvant treatment plan.

At postoperative re-evaluation, all surgical incisions were healing appropriately without evidence of infection, dehiscence, or excessive inflammation. Although gross disease had been successfully removed, incomplete surgical margins in the right medial distal thigh and right caudal hock lesions, together with very narrow margins in the right cranial tibial and right medial proximal thigh lesions, raised concern for residual microscopic disease in the right pelvic-limb surgical beds. Systemic treatment was considered but was not elected. Adjuvant IsoPet^®^ Precision Radionuclide Therapy (PRnT™) was recommended to treat suspected residual microscopic disease while preserving the function of the affected limb.

## 4. Adjuvant IsoPet^®^ Precision Radionuclide Therapy

Following postoperative healing, the treatment was performed 21 days later approximately three weeks after limb-sparing surgery. The treatment utilized yttrium-90 (Y-90) phosphate hydrogel. Treatment planning used a patient-specific, grid-based IsoPet^®^ margin protocol intended to treat suspected residual microscopic disease along each surgical scar and adjacent tumor bed. Prescribed activity was calculated from the measured length of each excised-tumor incision so that the planned dose was 400 Gy along the scar line and 200 Gy in the parallel rows adjacent to the scar. Using the IsoPet^®^ treatment plan, a total activity of 8.46 mCi (313 MBq) corresponding to an activity concentration of 1.8 mCi/mL was administered in a total volume of 4.7 mL. Injections followed the manufacturer margin-treatment grid. To achieve a uniform dose distribution along the center of the scar, 0.1 mL was injected approximately every 8 mm (nominal spacing per the IsoPet^®^ protocol; minor adjustments were made to accommodate exact scar length and local anatomy) in the subcutaneous/peri-incisional tissues of the prior tumor bed using a 1-mL syringe and 25-gauge needle. Parallel rows 0.8 mm lateral to each edge of the scar received 0.05 mL at approximately 8 mm intervals.

The grid-based treatment-planning approach was used for the right hock, medial thigh, and proximal thigh surgical beds. At the right cranial tibial site, six scar-line injections were administered. Parallel-row injections were not performed at this site because of limited soft tissue. Therefore, the planned parallel-row component of the 400 Gy/200 Gy dose distribution was not delivered at this site. The treatment plan was intended to distribute the prescribed activity along the surgical incisions and adjacent tissues. The resulting peri-incisional map comprised multiple small-volume deposits rather than a single bolus. The surgical bed of the completely excised metastatic right popliteal lymph node was not included in the Y-90 treatment field.

Site-level volumes were: right hock (5-cm incision), nine 0.1 mL scar-line injections (0.9 mL) plus 16 injections of 0.05 mL lateral and medial to the incision; right cranial tibia (4-cm incision), six 0.1 mL scar-line injections (0.6 mL); right medial thigh (4-cm incision), six 0.1 mL scar-line injections (0.6 mL) plus 10 injections of 0.05 mL lateral and medial to the incision (0.5 mL); right proximal thigh (4-cm incision), seven 0.1 mL scar-line injections (0.7 mL) plus 12 injections of 0.05 mL lateral and medial to the incision (0.6 mL). Imaging-based or Monte Carlo verification of the delivered dose was not performed after injection; planning relied on this length-based grid calculation.

The procedure was performed without intraoperative complications, and radiation safety measurements were performed prior to discharge following the institutional protocol (Figure 1). The patient recovered uneventfully from anesthesia and was discharged on the day of treatment with routine postoperative instructions including monitoring the incision, activity restriction, and radiation safety recommendations. A standardized owner questionnaire completed 24 h after treatment revealed only a mild reduction in appetite during the immediate postoperative period. Appetite improved spontaneously within the following day, and no vomiting, diarrhea, changes in urination or defecation, or deterioration in mobility were reported. The owners also reported no redness, swelling, discharge, or discomfort associated with the treatment sites.

On re-evaluation 11 days after Y-90 treatment, all surgical incisions were healing well, with only mild localized irritation noted over the proximal medial thigh incision. There was no evidence of wound infection, tissue necrosis, delayed healing, or other treatment-related complications. Mild soft tissue swelling was observed around the right hock treatment site at the subsequent visit 29 days after Y-90 treatment, which was consistent with expected postoperative tissue response and did not require additional intervention (Figure 2). Importantly, there was no palpable evidence of local tumor recurrence noted at any of the previously treated surgical sites.

The patient was re-evaluated again 64 days after Y-90 treatment, on 23 January 2026. Examination of the right pelvic limb continued to show satisfactory healing, with no clinically detectable local recurrence at the treated sites. However, during this visit the owners reported persistent left-sided otitis externa that had failed to resolve despite multiple courses of topical therapy including Otipak^®^, Easotic^®^ and BNP ointment. Physical examination confirmed chronic otitis externa with associated pyotraumatic dermatitis secondary to persistent scratching. Due to the chronicity of the otic disease and its poor response to medical treatment, advanced imaging was recommended to investigate the possibility of an underlying structural abnormality.

Seventy-four days after IsoPet^®^ treatment, computed tomography of the head and cervical region was performed on 2 February 2026. Imaging revealed chronic bilateral otitis externa, with severe mineralization of the external ear canals and tympanic bullae. In addition, an aggressive expansile lesion involving the right C5 vertebra was identified. The lesion was characterized by extensive osteolysis, destruction of the vertebral body and associated processes, and extension into the vertebral canal with associated extradural spinal cord compression. The lesion was highly suspicious for an aggressive neoplastic process; however, no histopathological confirmation was obtained. The relationship between this lesion and the previously treated pelvic limb neoplasia could not be definitively established. There was no clinically detectable local recurrence at the original treatment sites.

At re-evaluation 92 days after Y-90 treatment, the owner reported that the pet was doing well at home and continued to eat and drink normally. Neurological examination remained normal. Examination of the previous right pelvic limb surgical sites again showed no evidence of tumor regrowth. Medical management was continued for the cervical lesion and associated clinical signs, including gabapentin, prednisone, diazepam, and meclizine.

At the final documented re-evaluation on 20 March 2026 at Capital Veterinary Specialists, 120 days after Y-90 treatment, the previous right pelvic-limb treatment sites were examined by visual inspection and palpation, and no clinically detectable local recurrence was identified. A new small nodule was noted on the left distal pelvic limb; however, further diagnostic evaluation was not pursued at the owner’s request, and no definitive diagnosis was established. According to the referring veterinarian’s records, the patient was subsequently euthanized by the referring veterinarian on 9 June 2026 because of declining quality of life associated with progressive neurological weakness, ataxia, cervical pain, and quadriparesis, 201 days after Y-90 treatment and 222 days after surgery (Table 1).

## 5. Discussion

The present case describes the management of multifocal poorly differentiated carcinoma involving the right pelvic limb using a limb-sparing surgical approach followed by adjuvant IsoPet^®^ Precision Radionuclide Therapy (PRnT™). The case is clinically relevant because the multifocal distribution and distal limb location made conventional wide excision challenging, while the histopathological findings demonstrated incomplete margins and regional lymph node metastasis. In this setting, treatment was directed toward achieving gross tumor removal while preserving limb function, followed by localized treatment of the surgical beds at risk for residual microscopic disease. Although the right popliteal lymph node contained metastatic carcinoma, it was completely excised, and its surgical bed was not included in the Y-90 treatment field.

Complete surgical excision was pursued as the primary local treatment in this case. However, achieving wide margins in the distal limb can be difficult because of limited soft tissue and the proximity of important anatomical structures [13,15]. Incomplete histological margins remain a recognized consequence of planned narrow excision of appendicular soft tissue sarcomas, particularly when anatomical constraints limit the ability to achieve wider margins [14]. Postoperative radiation therapy is therefore frequently considered when microscopic residual disease remains following surgery [2,3,12]. In the present case, four grossly evident masses were removed while preserving the affected limb, but histopathology demonstrated incomplete margins in selected lesions. The right popliteal lymph node also contained metastatic carcinoma, further supporting the presence of biologically aggressive disease.

The major therapeutic consideration following surgery was the risk of residual microscopic disease. Conventional external beam radiation therapy represents an established option for incompletely excised canine soft tissue sarcomas and can provide prolonged local control [2,3,12]. However, conventional radiation therapy requires multiple treatment fractions and repeated anesthetic episodes. This, combined with the high cost of radiation therapy, can be particularly challenging when multiple surgical beds are distributed along a distal limb.

Y-90 hydrogel provides a different approach to localized radiation delivery. The treatment utilizes Y-90 phosphate particles incorporated within an injectable hydrogel. Y-90 is a beta-emitting radionuclide, and the relatively short tissue penetration of its emitted particles allows radiation to be concentrated around the treatment site. Experimental investigations of Y-90–polymer composites have demonstrated localized retention of the radioactive material and support the feasibility of delivering high-dose radiation directly to solid tumor tissues [6,7].

An important clinical observation was the patient’s postoperative tolerance. Only mild transient reduction in appetite was reported immediately following treatment, and no significant gastrointestinal signs or clinically important local tissue complications were documented. Serial examinations demonstrated satisfactory wound healing, with only mild localized irritation and swelling during follow-up. These findings are consistent with the favorable short-term tolerability reported with localized Y-90 treatment, although conclusions regarding the safety profile of IsoPet^®^ cannot be drawn from a single patient.

The most clinically relevant local observation was the absence of palpable regrowth at the original right pelvic-limb treatment sites during the 120-day documented follow-up period. This finding followed combined limb-sparing excision and adjuvant Y-90; because all grossly visible tumors had already been removed and there was no control case, it cannot be attributed specifically to IsoPet^®^. Follow-up was limited, and no post-treatment advanced imaging or biopsy of the treated beds was performed, so microscopic residual disease cannot be excluded. Therefore, these findings are described as no clinically detectable local recurrence rather than as established local control or treatment efficacy.

Subsequently, an aggressive osteolytic lesion was identified outside the original treatment field. Computed tomography performed because of persistent otitis demonstrated an aggressive lesion involving the right C5 vertebra with extension into the vertebral canal and extradural spinal cord compression. The lesion was highly suspicious for an aggressive neoplastic process; however, no histopathological confirmation was obtained. The relationship between this lesion and the previously treated pelvic limb neoplasia could not be definitively established. The development of an aggressive osteolytic lesion suspicious for neoplasia outside the original treatment field illustrates that the absence of clinically detectable local recurrence at the treated limb sites does not indicate control of disease elsewhere.

The case also highlights the distinction between local treatment efficacy and overall oncologic outcome. IsoPet^®^ was administered to address suspected residual microscopic disease within the surgical bed. No clinically detectable local recurrence was observed at the treated sites during the 120-day documented follow-up period after combined surgery and Y-90. That observation should not be interpreted as proof of local efficacy, nor as evidence that IsoPet^®^ prevented metastatic or systemic progression. A new small nodule was identified on the left distal pelvic limb at the final documented examination, 120 days after Y-90 treatment. Further diagnostic evaluation was not pursued at the owner’s request; therefore, the nature of the nodule and its relationship to the original neoplasia remain undetermined. This finding highlights the importance of distinguishing the absence of clinically detectable recurrence at treated sites from the development of new lesions at other locations. Without a definitive diagnosis, the nodule cannot be classified as metastatic disease or a new primary lesion.

Several limitations should be considered. First, this is a single case, and the findings cannot be generalized to other dogs or tumor types. Second, the discrepancy between the initial punch biopsy and definitive excisional histopathology prevented complete characterization of the tumor’s histologic origin. Immunohistochemical characterization was not performed because of cost considerations and because additional characterization was not expected to alter the treatment plan. Third, local control was assessed clinically rather than through routine post-treatment advanced imaging or repeat biopsy, and therefore microscopic residual disease cannot be excluded. Finally, the cervical lesion was not histologically confirmed, preventing definitive determination of whether it represented metastasis from the original tumor or an unrelated primary neoplasm. The dog subsequently developed progressive neurological weakness, ataxia, cervical pain, and quadriparesis, resulting in a decline in quality of life and euthanasia. No necropsy was performed; therefore, the underlying cause of the neurological progression and the nature of the cervical lesion could not be confirmed postmortem.

Despite these limitations, the case adds to the emerging clinical experience with Y-90 hydrogel as an adjunctive treatment following limb-sparing surgery. In particular, it demonstrates the practical application of localized Y-90 therapy to multiple postoperative treatment beds in a large dog with anatomically challenging distal limb disease. Further prospective studies involving larger numbers of patients, standardized dosimetry, objective imaging-based assessment of local control, histopathological confirmation where appropriate, and longer follow-up are required to determine the efficacy and appropriate clinical indications of IsoPet^®^ in veterinary oncology.

In this case, Y-90 phosphate hydrogel was technically feasible and well tolerated following limb-sparing surgery for poorly differentiated carcinoma of the right pelvic limb with incomplete surgical margins. No clinically detectable local recurrence was observed at the treated limb sites during the 120-day documented follow-up period. Because gross disease had been surgically removed and this was a single case without a comparator, the observed outcome cannot establish the independent efficacy of Y-90 hydrogel. Further clinical evaluation is required to determine its efficacy and appropriate application in veterinary oncology.

## Figures and Tables

**Figure 1:**
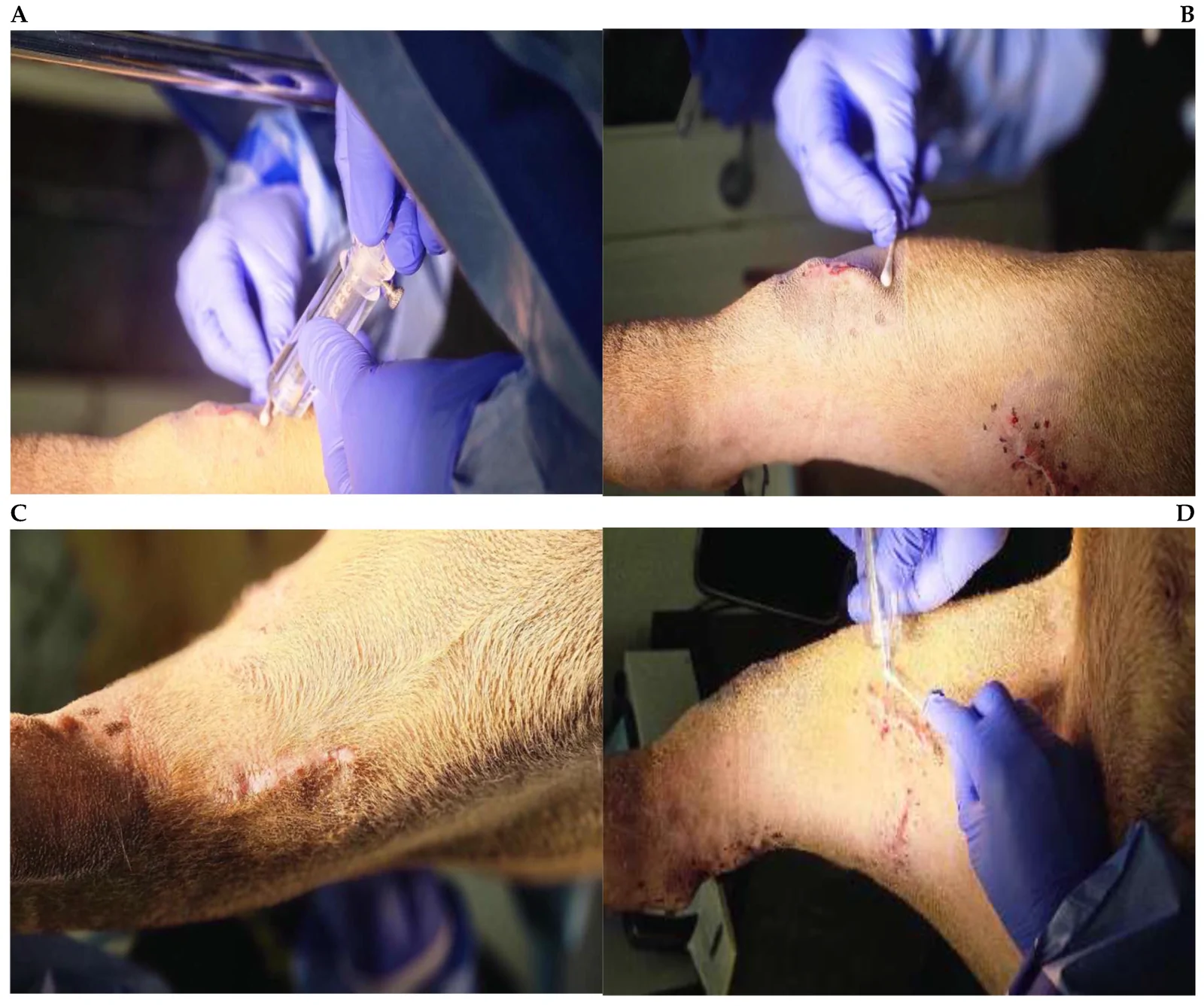
Peri-incisional administration of yttrium-90 (Y-90) phosphate hydrogel (IsoPet^®^) following limb-sparing excision of multifocal neoplasia of the right pelvic limb. Panels show Y-90 hydrogel injection at the following sites: (**A**) Right medial distal thigh; (**B**) right medial proximal thigh; (**C**) right caudal hock; and (**D**) right lateral cranial tibia.

**Figure 2:**
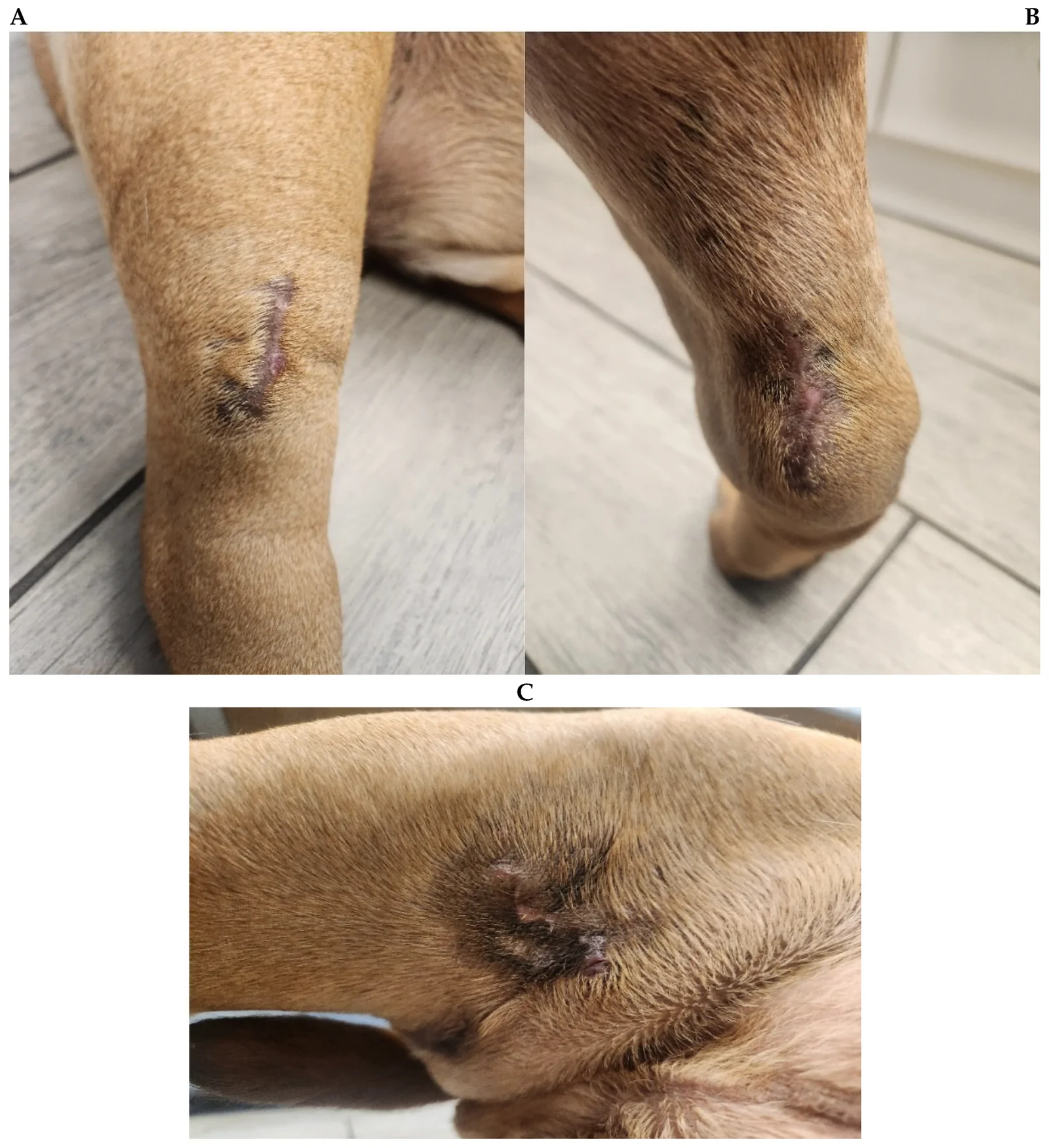
Clinical appearance of the treated right pelvic limb surgical sites at the 29-day post-treatment fol-low-up following IsoPet^®^ Precision Radionuclide Therapy. (**A**) Right medial thigh; (**B**) right cranial tibia; and (**C**) right caudal hock.

**Table 1:** Chronological timeline illustrating the diagnostic workup, surgical management, adjuvant IsoPet^®^ Precision Radionuclide Therapy (PRnT™), follow-up evaluations, and clinical outcome.

Date	Clinical Event
21 April 2025	Annual wellness examination revealed new right hind limb lameness, progressive pain and decreased range of motion of the right hip.
15 August 2025	Multiple subcutaneous masses identified along the right pelvic limb. Fine-needle aspiration performed; inflammatory cytology obtained, but neoplasia could not be excluded.
9 September 2025	Progressive enlargement of existing masses despite medical therapy.
29 September 2025	Punch biopsies performed. Histopathology identified Grade II soft tissue sarcoma in sampled lesions.
October 2025	Referred to Capital Veterinary Specialists for surgical oncology evaluation.
30 October 2025	Limb-sparing excision of four right pelvic limb masses and right popliteal lymph node performed.
Postoperative histopathology	Poorly differentiated carcinoma involving all excised masses with metastatic involvement of the right popliteal lymph node; incomplete surgical margins identified.
20 November 2025	Adjuvant IsoPet^®^ Precision Radionuclide Therapy (PRnT™) administered (8.46 mCi Y-90 (313 MBq); 66 peri-incisional injections).
1 December 2025	Recheck 11 days after Y-90 treatment. Mild irritation of the proximal medial right thigh scar was noted; otherwise, the physical examination was normal. Continued monitoring of the injection sites was recommended.
19 December 2025	Recheck 29 days after Y-90 treatment. The owner reported swelling around the hock injection site, but the dog was otherwise doing well at home. Neurological examination was normal, and no regrowth was identified at the previous right hind-limb surgical sites. The incision was healed.
23 January 2026	Recheck 64 days after Y-90 treatment. The neurological examination was normal, and no evidence of regrowth was identified at the previous right hind-limb surgical sites. Left-sided otitis externa and pyotraumatic dermatitis of the left buccal region were noted.
2 February 2026	CT examination 74 days after Y-90 treatment revealed an aggressive osteolytic lesion involving the right C5 vertebra, with extradural spinal cord compression. The lesion was highly suspicious for neoplasia but was not histologically confirmed. No clinically detectable local recurrence was identified at the original treatment sites.
20 February 2026	Re-evaluation, 92 days after Y-90 treatment; no evidence of regrowth at previous right hind-limb surgical sites. Medical/palliative management continued.
12 March 2026	Clinical update 112 days after Y-90 treatment. Increased morning lethargy and head-down posture; gabapentin increased to 600 mg/day with reported improvement.
20 March 2026	Final documented local examination 120 days after Y-90 treatment; doing well at home with no clinically detectable right hind-limb regrowth. A new small nodule was noted on the left distal pelvic limb; further diagnostic evaluation was not pursued at the owner’s request, and no definitive diagnosis was established.
9 June 2026	Euthanasia performed by the referring veterinarian because of neurological weakness, ataxia, cervical pain, and quadriparesis, with declining quality of life; 201 days after Y-90 treatment and 222 days after surgery.

## Data Availability

The original contributions presented in this study are included in the article. Further inquiries can be directed to the corresponding author.
